# Viral and Bacterial Communities Collaborate through Complementary Assembly Processes in Soil to Survive Organochlorine Contamination

**DOI:** 10.1128/aem.01810-22

**Published:** 2023-02-21

**Authors:** Shujian Yuan, Ville-Petri Friman, Jose Luis Balcazar, Xiaoxuan Zheng, Mao Ye, Mingming Sun, Feng Hu

**Affiliations:** a Soil Ecology Lab, Key Laboratory of Plant Community, Jiangsu Collaborative Innovation Center for Solid Organic Waste Resource Utilization & Jiangsu Key Laboratory for Solid Organic Waste Utilization, Nanjing, China; b Department of Biology, University of York, York, United Kingdom; c Department of Microbiology, University of Helsinki, Helsinki, Finland; d Catalan Institute for Water Research (ICRA), Girona, Spain; e University of Girona, Girona, Spain; f College of Environmental and Resource Sciences, Zhejiang University, Hangzhou, China; g Key Laboratory of Soil Environment and Pollution Remediation, Institute of Soil Science, Chinese Academy of Sciences, Nanjing, China; University of Nebraska-Lincoln

**Keywords:** organochlorine pesticides, microbial community assembly, virus, soil, virus-host interactions

## Abstract

The ecological drivers that direct the assembly of viral and host bacterial communities are largely unknown, even though viral-encoded accessory genes help host bacteria survive in polluted environments. To understand the ecological mechanism(s) of viruses and hosts synergistically surviving under organochlorine pesticide (OCP) stress, we investigated the community assembly processes of viruses and bacteria at the taxon and functional gene levels in clean and OCP-contaminated soils in China using a combination of metagenomics/viromics and bioinformatics approaches. We observed a decreased richness of bacterial taxa and functional genes but an increased richness of viral taxa and auxiliary metabolic genes (AMGs) in OCP-contaminated soils (from 0 to 2,617.6 mg · kg^−1^). In OCP-contaminated soils, the assembly of bacterial taxa and genes was dominated by a deterministic process, of which the relative significance was 93.0% and 88.7%, respectively. In contrast, the assembly of viral taxa and AMGs was driven by a stochastic process, which contributed 83.1% and 69.2%, respectively. The virus-host prediction analysis, which indicated *Siphoviridae* was linked to 75.0% of bacterial phyla, and the higher migration rate of viral taxa and AMGs in OCP-contaminated soil suggested that viruses show promise for the dissemination of functional genes among bacterial communities. Taken together, the results of this study indicated that the stochastic assembly processes of viral taxa and AMGs facilitated bacterial resistance to OCP stress in soils. Moreover, our findings provide a novel avenue for understanding the synergistic interactions between viruses and bacteria from the perspective of microbial ecology, highlighting the significance of viruses in mediating bioremediation of contaminated soils.

**IMPORTANCE** The interaction between viral communities and microbial hosts has been studied extensively, and the viral community affects host community metabolic function through AMGs. Microbial community assembly is the process by which species colonize and interact to establish and maintain communities. This is the first study that aimed to understand the assembly process of bacterial and viral communities under OCP stress. The findings of this study provide information about microbial community responses to OCP stress and reveal the collaborative interactions between viral and bacterial communities to resist pollutant stress. Thereby, we highlight the importance of viruses in soil bioremediation from the perspective of community assembly.

## INTRODUCTION

Bacterial viruses (here referred to as viruses), which are the most abundant and diverse entities on earth, play a key role in the ecological and evolutionary processes of microbial communities by directly killing hosts or integrating into host genomes without lysing them (1–6). During lytic infection, viruses directly lyse host bacterial cells in a short period, exerting “top-down” regulation on bacterial communities (7). However, viruses can also integrate into their host’s bacterial genome and then redirect host bacterial metabolic activities through virus-encoded auxiliary metabolic genes (AMGs) (8). These virus-encoded AMGs have been widely recognized as impacting diverse metabolic activities in the soil, such as nutrient element transformation, energy metabolism, and contaminant biodegradation (9–11). For instance, the organochlorine pesticide-degrading gene L-DEX, atrazine-degrading gene *trzN*, and various chromium (Cr)-detoxifying genes have been identified as virus-encoded AMGs in contaminated soils (4, 11, 12). Moreover, a greater diversity of virus-encoded AMGs has been shown to help bacterial hosts survive in contaminated environments (11). Therefore, it is essential to explore the role of viral communities as ecological drivers that shape microbial communities in contaminated soils.

Microbial community assembly is the process of species colonization and interaction to establish and maintain local communities. This process, which occurs through continuous migration from the regional species pool, is driven by stochastic and deterministic processes (13). The deterministic factors include environmental filtering of abiotic conditions and biotic interactions (e.g., competition, exploitation, mutualism, predation, and host filtering), while the stochastic factors consist of unpredictable birth, death, reproduction, and diversification (14). The process of bacterial community assembly varies according to the types and extent of pollution in soils. A previous study found that the assembly of rare and abundant bacterial communities was dominated by different processes in oil-contaminated soils. Specifically, while the abundant bacterial subcommunity was influenced mainly by edaphic factors (i.e., deterministic processes), the assembly of the rare subcommunity was dominated by stochastic processes (15). In another study, both deterministic and stochastic processes drove the succession of a bacterial community under polychlorinated biphenyls stress, indicating the dynamic nature of bacterial community assembly (16). Recent studies have explored the assembly of viral communities to understand the comprehensive response of microbial communities to environmental stresses. Danczak et al. (17) revealed that the viral community was influenced by different assembly processes with time in fractured shale ecosystems and that the processes of viral and bacterial community assembly were interrelated. However, compared with bacterial community assembly processes, the factors that drive the assembly of viral communities and their interactions with bacterial communities in contaminated soils are less well understood.

Organochlorine pesticides (OCPs) are synthetic pesticides with broad applications in agricultural and chemical industries, which have been detected frequently worldwide in soils (18, 19). Due to their high toxicity and recalcitrance, soils contaminated by OCPs have been of great concern to human health and environmental security (20, 21). Organochlorine pesticide exposure exerts direct selection on soil bacterial communities. While bacterial taxa that are less resistant to OCPs become less abundant or disappear under heavy pollutant exposure, OCP-resistant bacterial taxa increase their abundance due to a competitive advantage (22). Our previous study suggested that viral communities played crucial roles in the bacterial survival of heavy OCP contamination (total pesticide content varying from 1,083.7 ± 40.4 to 4,595.8 ± 344.0 mg · kg^−1^) and the degradation of OCPs through virus-encoded AMGs in soils (11). However, the presence of genes is organism dependent, and viral communities are regulated by host population dynamics or phage defense systems. Therefore, we hypothesize that the assembly process at the taxonomic and functional levels of bacteria and virus in OCP-exposed soils was consistent and that bacterial community assembly was driven by a deterministic process, while viral communities were dominated by stochastic assembly under OCP exposure. Moreover, viral communities could enable the deterministic assembly of bacterial communities due to the potential benefits for the host bacteria.

In this study, we used a combination of metagenomics/viromics and bioinformatics approaches to explore how OCP exposure affects the composition of both bacterial and viral communities in a closed OCP-contaminated site in the Yangtze River Delta, China. Variance in the assembly processes of bacterial taxa and functional genes, as well as viral taxa and AMGs, was investigated. We applied the normalized stochasticity ratio to investigate the bacterial and viral community assembly process at the taxon and gene levels in OCP-contaminated soil. The dominant processes that drove the assembly of bacterial and viral communities in heavily OCP-stressed soils were deterministic and stochastic, respectively. Moreover, the stochastic assembly of viruses and AMGs was found to help maintain functional redundancy of the bacterial community. Taken together, our findings suggest the need to study assembly processes at multiple trophic levels to comprehensively understand the ecological drivers of microbial communities under contaminant exposure.

## RESULTS

### Bacterial profiles in clean and OCP-contaminated soils.

**FIG 1:**
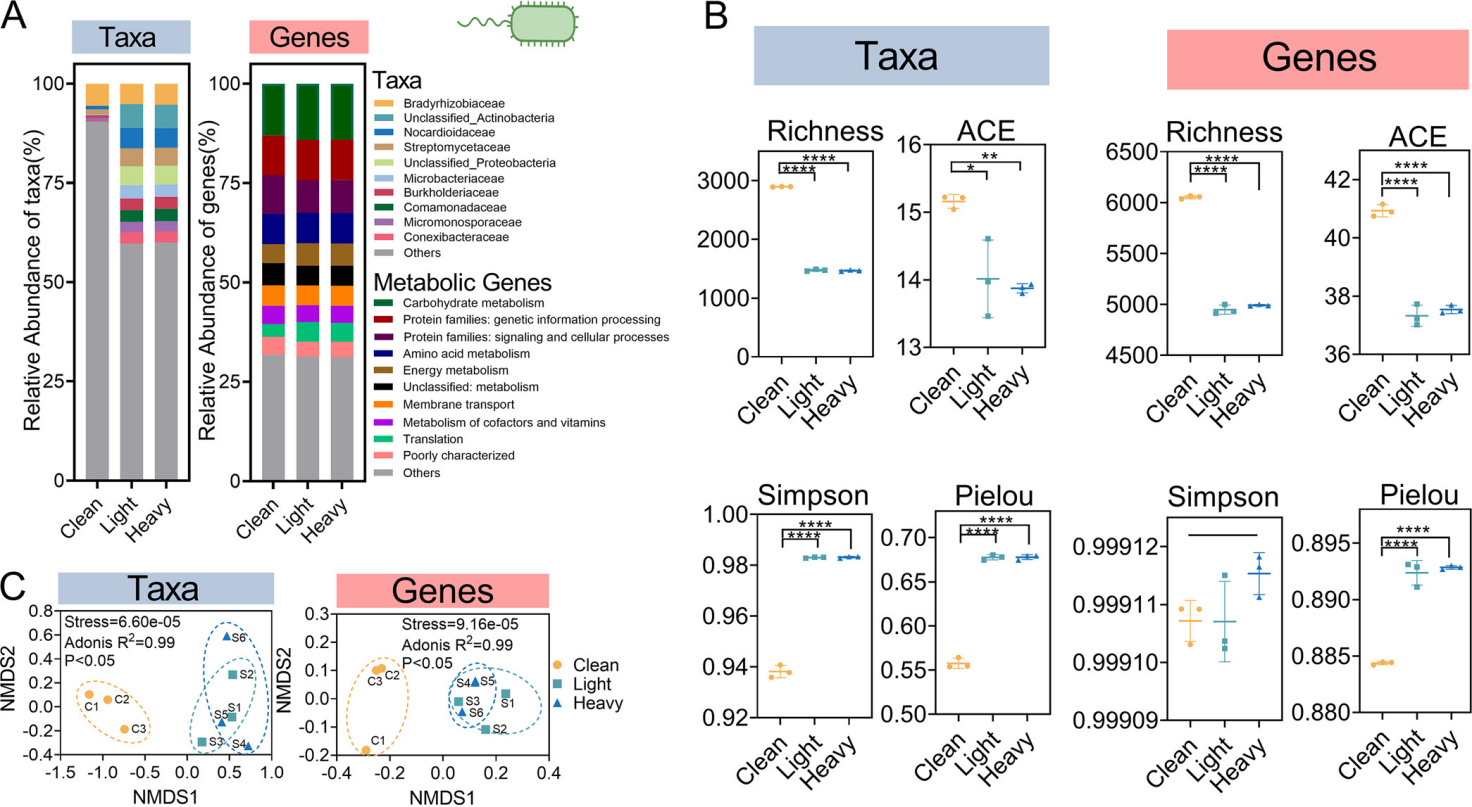
Differences in bacterial communities in clean and OCP-contaminated soils. (A) Relative abundance of the top 10 abundant bacterial families and the composition of functional gene classification based on the KEGG database in clean (C1 to C3) and OCP-contaminated (light, S1 to S3; heavy, S4 to S6) soils. “Others” represent the remaining taxa and functional genes. (B) Differences in bacterial taxa and functional gene alpha diversity in clean and OCP-contaminated soils. (C) NMDS analysis of bacterial taxa and functional genes in clean and OCP-contaminated soil. The asterisks indicates a significant difference between treatments with one, two, three and four asterisks indicate *P* < 0.1, 0.01, 0.001, 0.0001, respectively.

To determine the influence of OCP exposure on bacterial communities at different levels, we compared the relative abundance, alpha- and beta-diversity of bacterial taxa, and genes between clean and OCP-contaminated soil. Metagenomics analyses revealed that bacterial taxa and genes had distinct characteristics related to their relative abundances across the soils. We found distinct bacterial families in OCP-contaminated soils, such as unclassified *Actinobacteria* and unclassified *Proteobacteria* and *Comamonadaceae*, and OCP exposure was associated with increased relative abundances of *Nocardioidaceae* (clean, 0.9%; light, 5.1%; and heavy, 4.9%), *Streptomycetaceae* (clean, 1.3%; light, 4.6%; and heavy, 4.6%), *Microbacteriaceae* (clean, 0.2%; light, 3.4%; and heavy, 3.0%), *Burkholderiaceae* (clean, 0.7%; light, 3.0%; and heavy, 3.0%), *Micromonosporaceae* (clean, 0.8%; light, 2.7%; and heavy, 2.6%), and *Conexibacteraceae* (clean, 0.2%; light, 2.8%; and heavy, 2.8%) (Fig. 1A; see Table S1_taxonomy in the supplemental material). In addition, the relative abundances of organohalide-respiring bacteria (OHRBs), such as *Enterobacteriaceae*, *Desulfovibrio*, Pseudomonas (23), and *Dehalococcoides* (24), in OCP-contaminated soils were significantly higher than those in clean soils (one-way analysis of variance [ANOVA], *P* < 0.05). In contrast, the relative abundance of bacterial functional genes annotated by the Kyoto Encyclopedia of Genes and Genomes (KEGG) database remained stable across soils (Fig. 1A; Table S1_KEGG annotation). Lower richness (clean, 2,896.3; light, 1,475.7; and heavy, 1,468.3 for bacterial taxa; clean, 6,053.3; light, 4,948.0; and heavy, 4,991.3 for bacterial genes) and ACE indexes (clean, 15.2; light, 14.0; and heavy, 13.9 for bacterial taxa; clean, 40.9; light, 37.3; and heavy, 37.5 for bacterial genes) but higher Simpson’s (clean, 0.9; light, 1.0; and heavy, 1.0 for bacterial taxa; clean, 1.0; light, 1.0; and heavy, 1.0 for bacterial genes) and Pielou indexes (clean, 0.6; light, 0.7; and heavy, 0.7 for bacterial taxa; clean, 0.9; light, 0.9; and heavy, 0.9 for bacterial genes) of bacterial taxa and genes were detected in OCP-contaminated soils (Fig. 1B), suggesting decreased richness but increased evenness of bacterial communities due to OCP exposure. We also used nonmetric multidimensional scaling (NMDS) to study the Bray-Curtis distance of bacterial taxa and functional gene composition among treatments. Bacterial taxonomic and genetic composition clearly differed between clean and OCP-contaminated soils but not between light and heavy contaminated soils (Adonis analysis, *P* < 0.05) (Fig. 1C). Mantel analysis indicated a significant association between the composition of bacterial taxa and functional genes (Mantel test, *P* < 0.05).

### Viral profiles in clean and OCP-contaminated soils.

**FIG 2:**
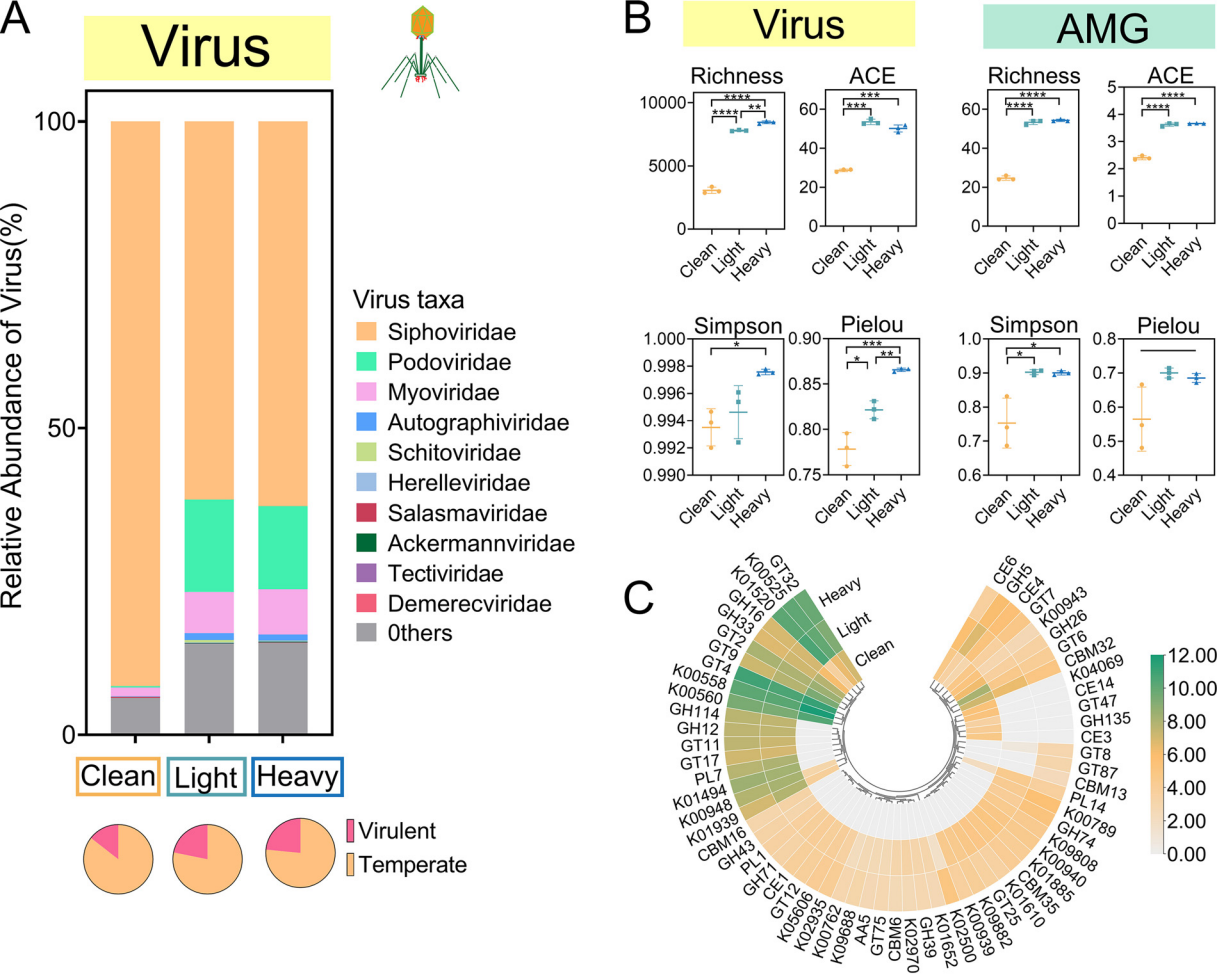
Differences in viral communities in clean and OCP-contaminated soils. (A) Relative abundance of the top 10 abundant viral families in clean (C1 to C3) and OCP-contaminated (light, S1 to S3; heavy, S4 to S6) soils. “Others” represent the remaining taxa. (B) Analysis of virus and AMG in clean and OCP-contaminated soil. (C) Differences in viral taxa and AMG alpha diversity in clean and OCP-contaminated soil. We used both KEGG and CAZy databases for viral AMG annotation, and the best hits (defined by bit score, default minimum threshold of 60) reported for each database were provided in a single output file to avoid overlap. The asterisks indicates a significant difference between treatments with one, two, three and four asterisks indicate *P* < 0.1, 0.01, 0.001, 0.0001, respectively.

*Siphoviridae* was the most abundant family in clean (92.0%) and OCP-contaminated soils (62.2% on average). OCP exposure was associated with increased relative abundances of *Podoviridae* (clean, 0.3%; light, 15.0%; and heavy, 13.5%), *Myoviridae* (clean, 1.4%; light, 6.7%; and heavy, 7.4%), and *Autographiviridae* (clean, 0.1%; light, 1.1%; and heavy, 1.0%) but decreased relative abundances of *Siphoviridae* (clean, 92.0%; light, 61.7%; and heavy, 62.7%) (see Table S2 in the supplemental material). Notably, *Schitoviridae* and *Demerecviridae* were detected only in OCP-contaminated soils (Fig. 2A). Specifically, the relative abundance of virulent viruses (average 22.6%) in OCP-contaminated soil was higher than that in clean soil (average, 14.5%) (one-way ANOVA, *P* < 0.05) (Fig. 2A), and the relative abundance of virulent viruses did not clearly differ between light and heavy contamination (one-way ANOVA, *P* > 0.05) (Fig. 2A). The alpha diversity of viral taxa indicated higher richness, ACE, Simpson, and Pielou indexes in OCP-contaminated soil (Fig. 2B). The viral taxa clustered together according to the OCP contamination gradient, but the viral taxa in clean and OCP-contaminated soils separated into two subgroups (see Fig. S1 in the supplemental material).

We annotated 3,277 viral AMG sequences in total to explore the effects of viruses on bacterial metabolism (see Table S3 in the supplemental material). We used both KEGG and CAZy databases for viral AMG annotation, and the best hits (defined by bit score, default minimum threshold of 60) reported for each database were provided in a single output file to avoid overlap. According to the KEGG database, most AMGs were annotated as “nucleotide metabolism,” “amino acid metabolism,” and “carbohydrate metabolism” (Fig. 2C). In addition, virus-encoded AMGs in OCP-contaminated soil (55 gene classes) linked to nutrient cycling (carbon [C], nitrogen [N], phosphorus [P], and sulfur [S]) and pesticide degradation are more diverse and exclusive than those in clean soil (33 gene classes) (Table S3). Specifically, the *cysH* gene associated with hydrogen sulfide metabolism and two genes associated with pesticide degradation (aldehyde dehydrogenase [ALDH] and l-2-haluric acid dehalogenase [EC: 3.8.1.2], which are responsible for the transformation of chlorobenzene and chloroalkene, respectively) were found only in OCP-contaminated soils. The virus-encoded AMGs cover a range of bacterial metabolic activities and pesticide degradation pathways, indicating that the AMGs can enhance the bacterial community nutrient metabolism and degradation of OCPs, thereby benefiting bacterial survival in OCP-contaminated soils. Similar to viral taxa, alpha diversity (richness, ACE, Simpson, and Pielou indexes) of viral AMGs was higher in OCP-contaminated soil than that in clean soil. Beta diversity analysis indicated that clean and OCP-contaminated soils had different AMG compositions (Fig. S1).

### Environmental factors drove the variation in bacterial and viral community structure.

To investigate the impact of abiotic factors on microbial communities, variance partitioning analysis (VPA) was used to determine the explanatory degree of different abiotic factors on bacterial and viral communities at the taxon and gene levels. Single abiotic factors and their combinations that can best explain the variance in bacterial and viral compositions were screened out (see Table S4 in the supplemental material). The explanatory degree of single abiotic factors was <20% on average, with pH making the greatest contribution to the variance in viral taxonomic and viral genetic composition (19.4% and 13.7%, respectively). The combination of pH, cation exchange capacity (CEC), and *m*-nitrochlorobenzene had the highest explanatory degree (97.3%) for bacterial taxonomic variance. Combinations of pH, CEC, and total phosphorus (TP) were associated most closely with viral taxa (70.7%); pH, CEC, and total nitrogen (TN) were associated most closely with bacterial genes (81.5%); and pH, TP, and *m*-nitrochlorobenzene were associated most closely with viral AMGs (78.6%; Fig. S2A). Together, the compositions of bacterial taxa, viral taxa, and their genes were simultaneously under the impact of multiple abiotic factors across soils.

### Community assemblies of bacterial taxa and genes.

**FIG 3:**
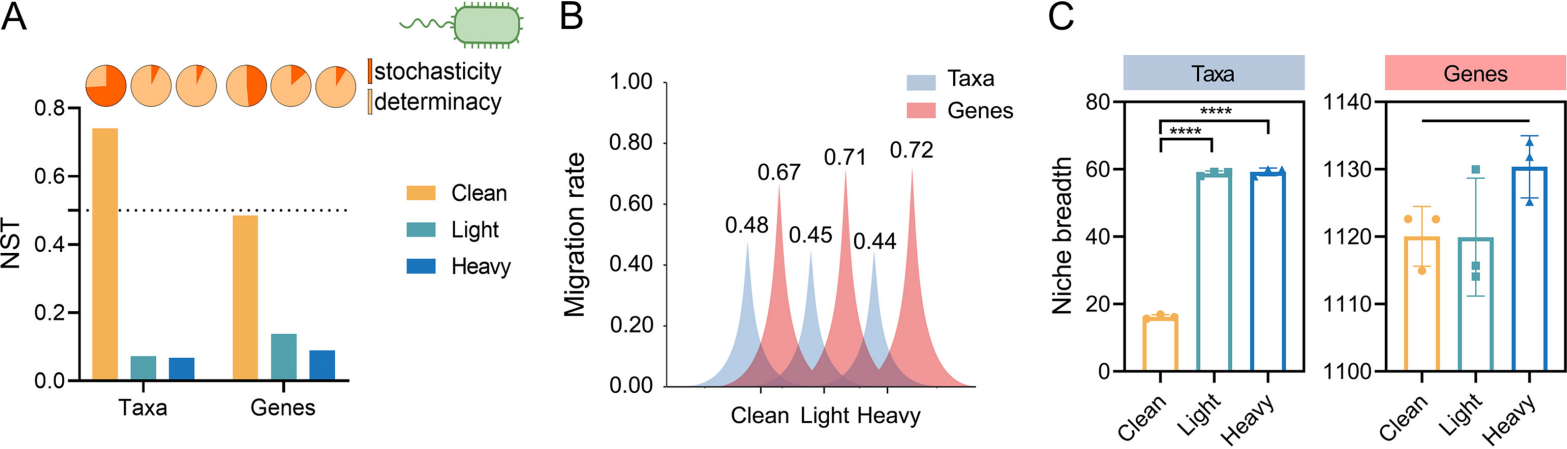
Assembly process of bacteria and genes in clean and OCP-contaminated soil. (A) The Normalized stochasticity ratio (NST) of bacterial taxa and functional genes in clean and OCP-contaminated soil. The NST of <0.5 represents the deterministic process, and the NST of >0.5 represents the stochastic process. (B) Difference in the migration rate of bacteria taxa and functional genes in clean and OCP-contaminated soil. (C) Differences in niche breadth of bacterial taxa and functional genes in clean and OCP-contaminated soil. The asterisks indicates a significant difference between treatments with one, two, three and four asterisks indicate *P* < 0.1, 0.01, 0.001, 0.0001, respectively.

The normalized stochasticity ratio (NST) was determined to investigate the community assembly processes affecting both bacterial and viral communities (25). The normalized stochasticity ratio reflects the relative importance of stochasticity in community assembly based on magnitude rather than on the significance of the difference between observed and null expectation as a quantitative measure of stochasticity. In this work, the NST was used to quantify the relative importance of deterministic (<50%) and stochastic processes (>50%) in community assembly (Fig. 3A). For bacterial community assembly, the relative importance of stochastic processes was 74.1%, 7.3%, and 6.8% in clean, light, and heavy contaminated soils, respectively, indicating that the relative importance of stochastic process decreased with elevated OCP contamination gradient in soils. Specifically, bacterial community assembly was dominated by stochastic processes in clean soil but driven by deterministic processes in OCP-contaminated soils. Similar to bacterial community assembly, the relative importance of stochastic processes on bacterial gene assembly decreased with increasing contamination levels, which were 48.5%, 13.7%, and 8.9% in clean, light, and heavy contaminated soils, respectively. Because the NST values were all lower than 50%, the bacterial gene assembly was dominated by deterministic processes in clean and OCP-contaminated soils, and the significance of deterministic processes increased with elevated OCP contamination.

Additionally, the migration rate of bacterial taxa in OCP-contaminated soils was significantly lower than that in clean soil (one-way ANOVA, *P* < 0.05) (Fig. 3B) but did not vary significantly between light and heavy contaminated soils (one-way ANOVA, *P* > 0.05) (Fig. 3B). Conversely, the migration rate of bacterial genes in OCP-contaminated soil was significantly higher than that in clean soil (one-way ANOVA, *P* < 0.05) (Fig. 3B) and could not be distinguished between light and heavy contaminated soils (one-way ANOVA, *P* > 0.05) (Fig. 3B). The niche breadth of bacterial taxa in OCP-contaminated soil was significantly higher than that in clean soil (16.2 ± 0.6) but did not differ significantly between light (58.8 ± 0.7) and heavy contaminated soils (59.2 ± 1.2) (one-way ANOVA, *P* > 0.05) (Fig. 3C). The niche breadth of bacterial genes in OCP-contaminated soil was higher than that in clean soil, but the differences in the niche breadth of bacterial functional genes in different contamination gradients were not significant (one-way ANOVA, *P* > 0.05) (Fig. 3C). OCP exposure exerted strong selection that decreased bacterial community mobility and increased the niche breadth of surviving bacteria on the bacterial community assembly process.

### Community assemblies of viral taxa and virus-encoded AMGs.

**FIG 4:**
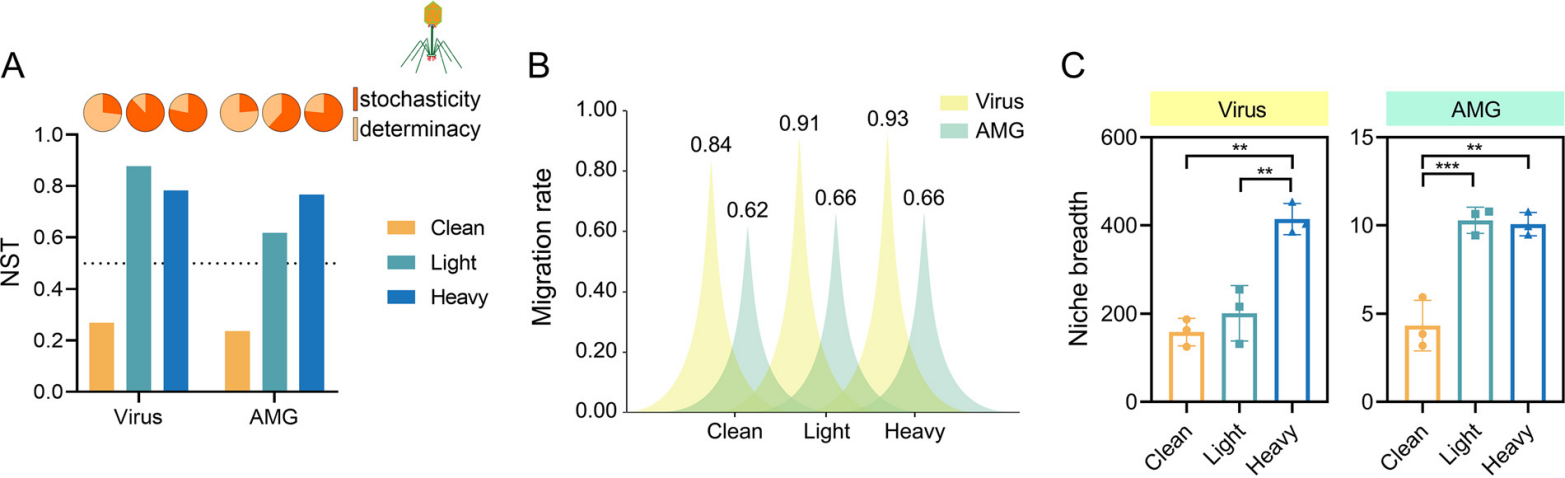
Assembly process of viral taxa and AMG in clean and OCP-contaminated soil. (A) The NST value of viral taxa and AMG in clean and OCP-contaminated soil. (B) Difference in the migration rate of viral taxa and AMG in clean and OCP-contaminated soil. (C) Differences in niche breadth of viral taxa and AMG in clean and OCP-contaminated soil. The asterisks indicates a significant difference between treatments with one, two, three and four asterisks indicate *P* < 0.1, 0.01, 0.001, 0.0001, respectively.

To investigate the impact of OCP exposure on the assembly of viral taxa and AMGs in soils, we also determined the NST, migration rate, and niche breadth of viral communities between clean and OCP-contaminated soils. The relative importance of stochastic process to viral taxa assembly was 26.8%, 87.8%, and 78.3% in clean, light, and heavy contaminated soils, respectively, indicating that OCP exposure enhanced the relative importance of stochastic processes in the assembly of viral communities. A similar trend was also observed for the assembly of viral AMGs. As a result, the assembly of viral taxa and AMGs was dominated by deterministic processes in clean soil but by stochastic processes in OCP-contaminated soils (Fig. 4A). Stochastic processes had strong effects on the viral community and AMG composition. The migration rate of viral taxa followed the order of clean soil < light contamination < heavy contamination (one-way ANOVA, *P* < 0.05) (Fig. 4B), indicating that OCP pollution significantly increased the migration rate of viral taxa in soil. Similarly, OCP exposure increased the migration rate of viral AMGs from 0.62 in clean soil to 0.66 in OCP-contaminated soils. The niche breadth of viral taxa and AMGs in OCP-contaminated soils was higher than in clean soil (one-way ANOVA, *P* < 0.05) (Fig. 4C). OCP exposure significantly increased the migration rate and niche breadth of viruses and AMG, while also inducing stochastic-dominated assembly processes.

### Putative virus-host linkage in clean and OCP-contaminated soils.

**FIG 5:**
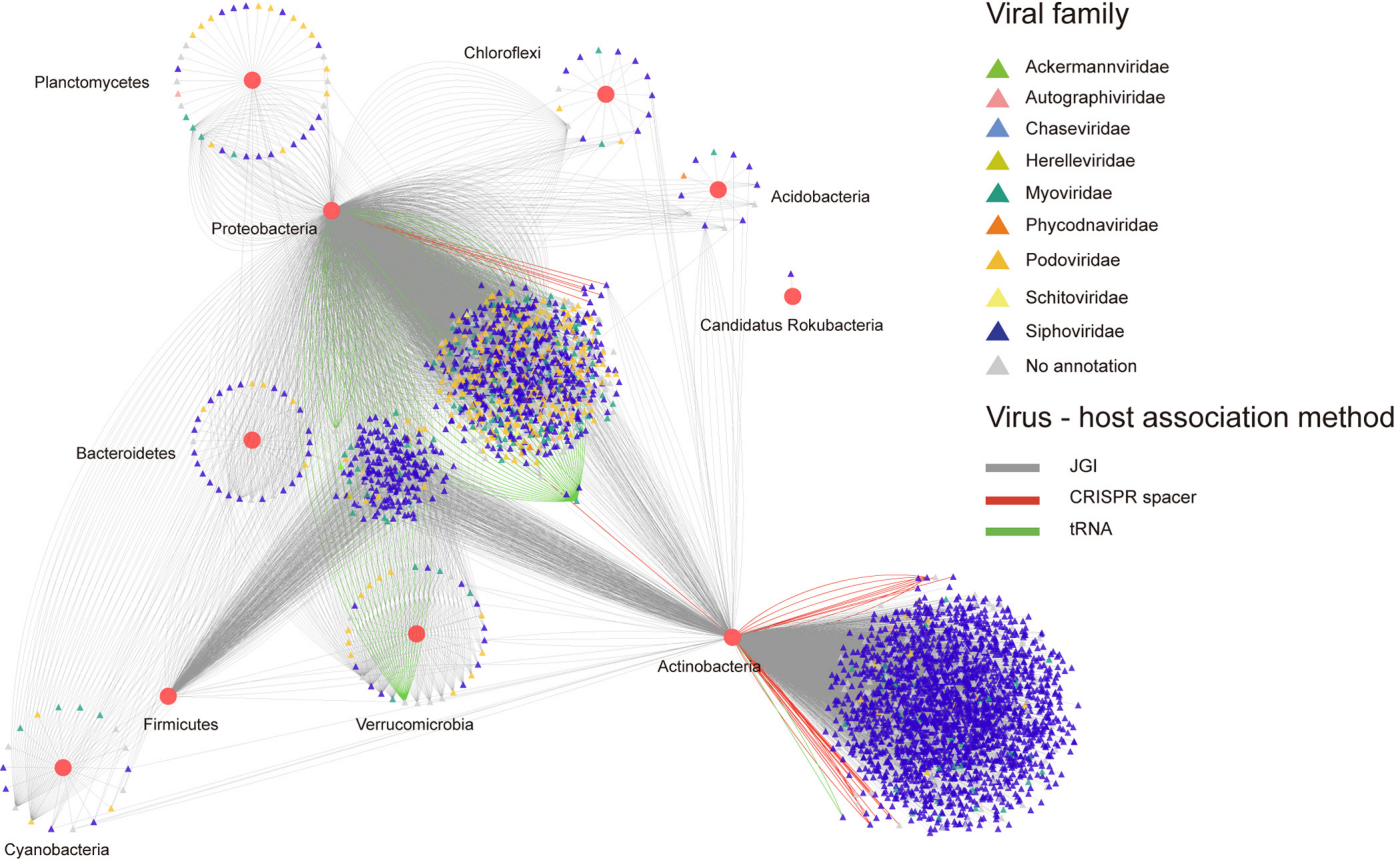
Virus-host associations based on tRNA matches, CRISPR spacer linkages and JGI database in clean and OCP-contaminated soils. Lines with different colors indicate the method of identifying virus-host associations (gray: JGI database; red: CRISPR spacer linkages; green: tRNA matches). Relative abundance of the top 10 abundant bacterial phyla hosts, red circles represent different bacterial phyla, and triangles of different colors represent viral contigs (family level).

We used three approaches, including tRNA sequence alignment, comparison of clustered regularly interspaced short palindromic repeats (CRISPR) spacers, and JGI database matching to explore virus-host linkage. Overall, 4,041 viral sequences were linked with 10,932 putative bacterial host sequences (belonging to 28 bacterial phyla) across the soils. *Siphoviridae* had the broadest host range, which was linked to 21 bacterial phyla, including the 10 most abundant bacterial phyla (Fig. 5). The remaining virus families consisted of *Podoviridae* (15 bacterial phyla), *Myoviridae* (13 bacterial phyla), *Autographiviridae* (3 bacterial phyla), *Phycodnaviridae* (2 bacterial phyla), *Herelleviridae* (2 bacterial phyla), *Schitoviridae* (1 bacterial phyla), *Chaseviridae* (1 bacterial phyla), and *Ackermannviridae* (1 bacterial phyla). *Phycodnaviridae*, *Herelleviridae*, *Schitoviridae*, *Ackermannviridae*, and *Chaseviridae* were detected only in OCP-contaminated soil. The relative abundance of the 10 most abundant bacterial phylum hosts were associated with 4,017 viral sequences (about 99.4% of all viral sequences matched the host). Of these hosts, the most abundant (*Proteobacteria*) was associated with 1,409 viral sequences (~34.9% of the overall viral sequences). Together, viruses exerted great impact on all dominant bacteria except for “*Candidatus* Rokubacteria,” which was infected only by one viral sequence. We defined viruses linking 10 bacterial phyla as broad-host viruses. Overall, *Siphoviridae* were classified as broad-host viruses in clean and OCP-contaminated soils, while *Podoviridae* and *Myoviridae* were considered broad-host viruses only in OCP-contaminated soils (see Table S5_Virus-host linkages in the supplemental material). Therefore, viruses in OCP-contaminated soil were linked to more diverse bacterial hosts than those in clean soil.

## DISCUSSION

### Bacterial genetic functions remained stable under OCP exposure.

In this work, although OCP exposure clearly changed the composition of bacterial taxa, it did not alter the composition of bacterial genes in soils (analysis of similarity [ANOSIM], *P* > 0.05) (Fig. 1A). The higher fluctuation of bacterial taxa could have occurred because the diversity of bacterial taxa in the soils was lower than that of bacterial genes (26). Functional redundancy of the bacterial community contributed to the stable composition of bacterial functional genes, regardless of OCP exposure. The decrease in the diversity of bacterial taxa could be associated with the disappearance of certain species that are sensitive to OCP stress in the soil (22), which could further impact the functional genes harbored by bacteria. Because of functional redundancy and frequent horizontal gene transfer (HGT) between bacteria, the impact of OCP exposure on bacterium-carried genes is commonly not as great as the impact on bacterial taxa (27, 28). In addition, the functional genes are often substitutable, and multiple genes perform the same function (29). New bases are generated at random sites of genes affected by gene mutation. Because of the degeneracy of codons (that is, multiple codons correspond to the same amino acid), codon changes caused by random gene mutation do not always affect the types of amino acids produced in the translation process; therefore, the function of the protein corresponding to the gene remains the same (30). Gene mutation (stochastic process) decreased the relative importance of the effects of OCP contamination stress on functional gene composition but did not completely change the gene functional classification. Overall, the gene functional classification remained stable under different OCP contamination stresses.

Viral life cycling also facilitated the functional redundancy of host bacteria. Viruses depend on bacterial hosts to complete their own reproduction, either by infecting and lysing host cells (lytic viruses) or by integrating their genes into the hosts’ genome without killing host cells (lysogenic viruses), allowing the virus to act as a vector for horizontal gene transfer between hosts (7, 8, 31). In this study, OCP exposure increased the proportion of lysogenic viruses, suggesting that a higher pool of viruses was involved in the horizontal transfer of functional genes. Moreover, OCP exposure increased the host range of viruses in soils, suggesting higher flexibility of the virus-host interaction and a greater probability of horizontal gene transfer between bacterial communities (11). The KEGG and CAZy annotations revealed the dominant AMG functional categories, including nucleotide metabolism, amino acid metabolism, and carbohydrate metabolism (Fig. 2C), which indicate that the viruses were capable of mediating the horizontal transfer of these functions between hosts, thereby contributing to the functional redundancy of bacterial communities under OCP-contaminated soils.

### Deterministic processes governed the assembly of bacterial communities in OCP-contaminated soils.

While community assembly of bacterial taxa has been studied extensively in soil ecosystems, bacterial genes remain less explored. Community assembly is influenced by both deterministic and stochastic processes. Deterministic processes include abiotic conditions (environmental filtering) and biotic interactions (such as competition, exploitation, mutualism, predation, and host filtering), while stochastic processes generally consist of drift, dispersal, and diversification (14, 32). We found that bacterial community assembly was dominated by stochastic processes in clean soils and dominated by deterministic processes in OCP-contaminated soils (Fig. 3A), which is consistent with the results of a previous study that revealed deterministic process drove the assembly of microbial communities in oil-contaminated soils (15). OHRB-mediated reductive dehalogenation removes halogens from various organohalogens and obtains the energy required for growth (23). OHRBs are also dominated by deterministic processes in OCP-contaminated soils, indicating the strong selective effect of OCPs on OHRBs. The intense OCP stress is associated with reduced bacterial taxa diversity (Fig. 1B), suggesting that selection is of major importance in community assembly.

The migration rate is the movement of species from a hypothetical species pool to a specific environment (33). The gradual decrease in bacterial taxa migration in response to OCP exposure indicated that the dispersal limitation decreased the significance of the stochastic process in the microbial assembly pattern in OCP-contaminated soil (34), which was consistent with the NST results. Additionally, niche breadth is related to species adaption to the environment, indicating the diversity and abundance of resources that species can use and their dispersal ability (35). In clean soils, weak environmental selective pressures and high taxa diversity resulted in great competition and small niche breadth of bacterial taxa. Conversely, OCP stress led to the disappearance of sensitive taxa, induced the community structure to be more homogeneous, and increased the number of niches available for surviving bacteria in the soil, thereby causing bacterial taxa in OCP-contaminated soils to have a broad niche breadth.

The assembly pattern of bacterial taxa and genes is consistent with the “competitive lottery mode” derived from the lottery hypothesis (36). This hypothesis holds that a specific niche in an ecosystem needs to be occupied by species with corresponding functional genes (the selection of functional genes is a deterministic process), but the niche will be occupied by bacterial species that arrive first (determination of the order of species occupying the ecological niche is stochastic). The functional gene migration rate is the horizontal transfer of genes between bacteria, and the increased gene migration rate in OCP-contaminated soil may be due to increased virus-mediated transduction as described above.

### Stochastic processes drove the assembly of viral communities in OCP-contaminated soils.

Currently, the assembly of viral communities under pollutant exposure is not well understood. Here, viromics analyses revealed that viral community assembly in the OCP-contaminated soil was dominated by stochastic processes (Fig. 4A). These findings are similar to those of a previous study that showed viral community assembly in salinity-stressed fractured shale ecosystems was driven by nondominant processes, including stochastic processes, such as weak selection, weak dispersal, diversification, and drift (17). The migration rate of the viral community increased with the degree of contamination, suggesting that the dispersal limitation was constrained and the relative importance of stochastic processes increased in OCP-contaminated soils (34). Because OCP exposure affected primarily viral communities through its effects on hosts, the hosts play an important role in the assembly of viral communities. In community ecology, the host can be regarded as a resource necessary for the virus survival, which is a special “niche” of viruses. Compared with viruses in clean soil, those in the OCP-contaminated soil had a broader host range and niche breadth (Fig. 4C). This finding indicates that there are more resources available for viruses and weaker competition within their community in OCP-contaminated soil, so the stochasticity of virus-host encounter rates becomes more important for virus assembly, further reducing the impact of deterministic process on viral community assembly. If most hosts are infected by various viruses (i.e., more niches overlap), the competition between viruses is pretty strong. Based on results of viral-host predictions, we screened 242 specialized host that were infected by only one viral sequence (45 in clean soil and 197 in OCP-contaminated soil), indicating that the virus in the OCP-contaminated soil had more specialized hosts and less niche overlap, which also reduce determinism in the viral community assembly (Table S5_Specialized host). Drift is more important when selection is weak and community size is small (14). Additionally, lytic viruses cannot be ignored when studying the assembly of viral communities. During the lysis process, the virus releases progeny, which results in a random change in the relative abundance of different virus species within the community. The increase in the proportion of lytic viruses in the OCP-contaminated soil may also facilitate the increased stochasticity of virus community assembly (Fig. 2A).

The ecological drivers that direct the assembly of viral and host bacterial communities are largely unknown, even though viral-encoded accessory genes help host bacteria to survive in polluted environments, such as arsenic-resistance gene *ars*C/*ars*M, organochlorine pesticide degradation gene L-DEX, and atrazine degradation gene *trz*N (4, 11, 37). Virus-encoded AMGs are a major pathway through which viruses become involved with and redirect host metabolic activities, and studying the assembly process of AMGs is of great importance for understanding how viruses affect host metabolism. The stochastic process of AMG assembly increased with the degree of OCP contamination, which is supported by the variations in AMG migration rate across different contamination gradients, as AMGs had a higher migration rate and lower dispersal limitation in OCP-contaminated soils. Genes are closely related to organisms, and generalist viruses provide greater possibilities for gene replication and expression than experts, so we examined the proportion of generalists or experts in AMG-containing viruses. The relative abundance of generalist AMG-containing phages is significantly higher than that of specialist AMG-containing phages in OCP-contaminated soils (Table S5_AMG-containing phages), and these generalist AMG-containing phages were dominated by stochastic assembly (Table S5_NST of generalists). Therefore, most AMGs in OCP-contaminated soils are stochastically assembled under the influence of the generalist AMG-containing phage assembly process. Considering that diversification is an evolutionary process that generates new genetic variation, higher AMG diversity in OCP-contaminated soils may represent a strong diversification effect (14). This increased diversification strengthened the effect of drift, further increasing the relative importance of the stochastic assembly process of AMGs in OCP-contaminated soils.

### Stochasticity of viral community assisted the bacterial community to relieve OCP stress.

**FIG 6:**
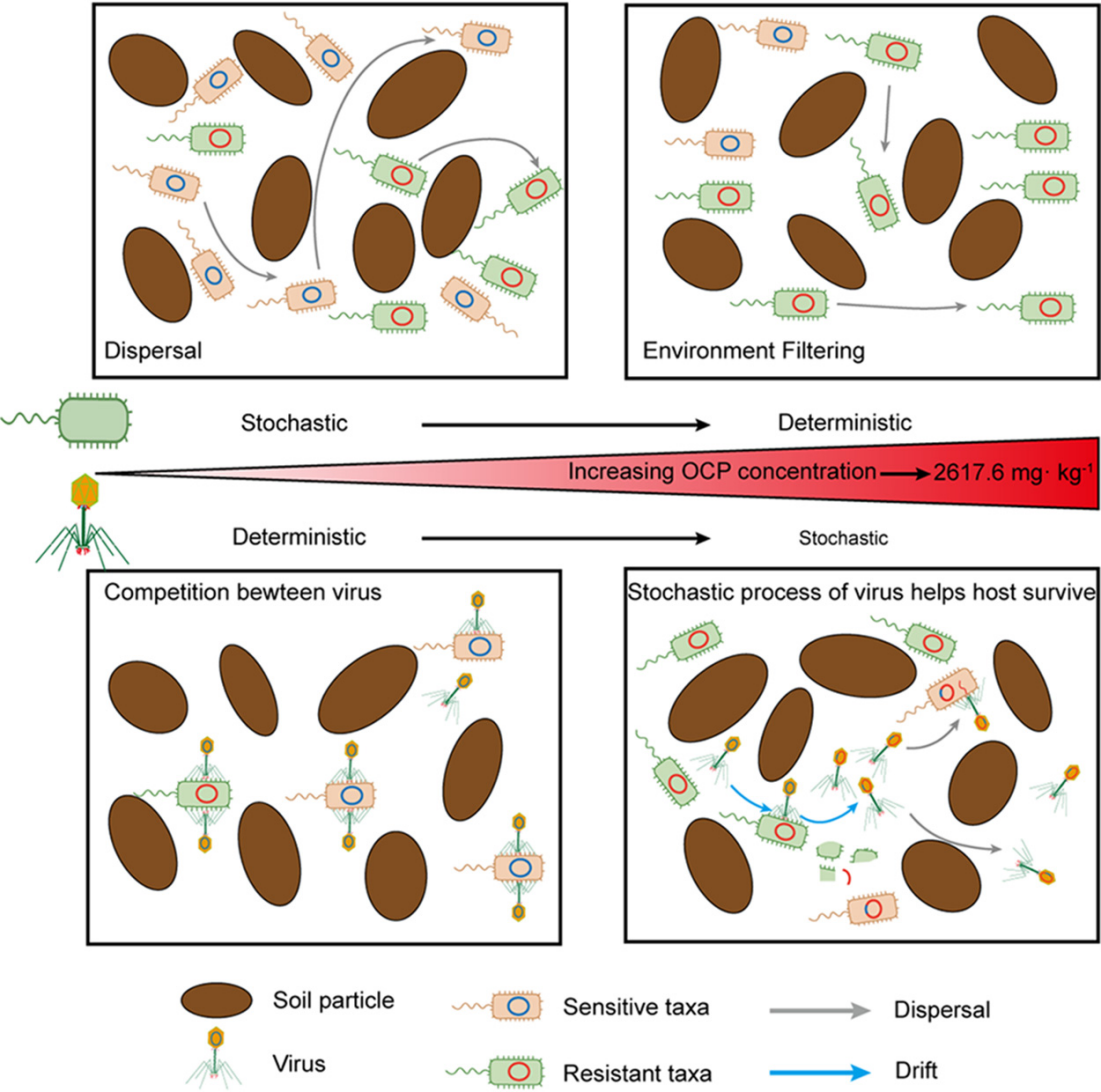
Conceptual model for bacterial and viral community assembly processes in clean and OCP-contaminated soils. The dispersal of bacteria in clean soil is strong, and community assembly is dominated by stochastic process; strong environmental filtration (deterministic process) dominates bacterial community assembly in OCP-contaminated soil. Virus community assembly is dominated by deterministic process in clean soil, and the competition of virus to host is an important factor affecting the deterministic process; virus community assembly is dominated by stochastic process (dispersal, drift, diversification) in OCP-contaminated soil, and the stochastic assembly process of the virus community helps the horizontal transfer of resistance genes among bacterial communities.

With the increase in OCP contents, the niche breadth of host bacteria and viruses that can infect the host and the relative abundance of host bacteria both increased (see Fig. S2B in the supplemental material), indicating that the host bacteria have a competitive advantage in OCP-contaminated soil. As described above, viruses facilitated the functional redundancy of host bacteria under OCP contamination. Nevertheless, the role of stochastic processes that dominate the assembly of viral taxa and AMGs needs further discussion. Our previous study revealed that the virus-carried L-2-haloacid dehalogenase gene (L-DEX) can be successfully expressed in Escherichia coli and can degrade l-2-haloacid pesticide precursors, thereby increasing the host ability to tolerate pesticide stress (11). Other virus-encoded stress-resistant AMGs were also detected and found to play promising roles in host adaptation to contaminant stress in soils, including chromium-resistant genes in chromium-contaminated slag sites and chlorohydrolase gene *trz*N in atrazine-contaminated soils (4, 12). In addition to antistress genes in the virus, the virus can also induce the acquisition of antistress genes by the bacterial community in many ways, and the stochastic assembly process of the virus and AMG also has a direct or indirect impact on bacterial communities in contaminated soil (Fig. 6) (38). The stochastic assembly process of viral communities, which consists of dispersal, diversification, and drift, might facilitate AMG dissemination between hosts. Drift represents changes in the relative abundance of taxa within a community caused by the inherent stochastic processes of unpredictable birth, death, reproduction, and diversification (14). The increased migration rate of viruses accelerates virus-mediated horizontal gene transfer (HGT) between bacterial hosts, which increases the dissemination of resistant genes within bacterial communities. Virus-mediated HGT plays an important role in bacterial ecology and evolution, which allows bacteria to evolve rapidly and to adapt to changing environmental conditions. Viruses can transfer genetic material between bacteria through generalized, specialized, and lateral transduction, and the widely detected viral AMGs appear to be the result of phages acquiring host metabolic genes through HGT events (39). Viruses can promote the occurrence of HGT in soil microbial communities under anthropogenic contaminated conditions, such as subinhibitory antibiotic contamination (40). In our previous work, a high number of polyvalent viruses carrying AMGs, which had more infectible bacterial hosts that could promote HGT of AMGs in bacterial communities, has been detected in OCP-contaminated soil compared with those in clean soil (11). We also detected the unique organochlorine pesticide degradation AMGs encoding L-DEX and aldehyde dehydrogenase (ALDH) and an increased abundance of viral AMGs linked to carbon, nitrogen, and sulfur metabolism in the OCP-contaminated soils (11). These AMGs involved in virus-mediated HGT were more diverse and transferred more frequently in OCP-contaminated soil than those in clean soil, suggesting that virus-mediated HGT could help the transfer of AMGs between hosts and expand the adaptation of bacterial communities to adverse conditions. Moreover, the diversification of viral AMGs can be considered a random mutation of genes. As a result, the stochastic dominated AMG assembly process in OCP-contaminated soil indicates a greater effect of diversification on AMG composition, thereby increasing the likelihood of variation in stress-resistant genes. Because of the coevolving and reciprocal interactions between organisms and their environment, viruses reprogram host metabolic activities through virus-carried AMGs to sustain a stable and favorable living environment. Taken together, the stochastic-process-dominated assembly of viral communities facilitates AMG dissemination among host bacteria, which helps to maintain the deterministic assembly of bacterial communities (Fig. 6). We suggest that viruses and their assembly processes provide a novel avenue for understanding the bioremediation of contaminated soils.

The key functional character of viruses is that they can positively alter host stress resistance in OCP-contaminated soils, which may have a tremendous impact on microbial community dynamics within relatively short time periods, while viruses can stably produce progeny within the host cell. Collectively, our results indicate that the viral community is a crucial biotic factor that cannot be ignored when studying the adaptation of bacterial communities to environmental stress.

### Conclusions.

We investigated the impact of OCP exposure on the taxonomic and genetic composition of viral and bacterial communities in soil using metagenomics and viromics approaches. The bacterial community composition varied significantly under different OCP-contaminated gradients, while the bacterial functional gene composition remained stable. As OCP contamination gradients increased, bacterial taxa and functional gene diversity decreased, while viral taxonomic and AMG diversity increased, resulting in the shift of bacterial community assembly from stochastic-dominant to deterministic-driven processes. In contrast, the assembly of both viral taxa and AMGs changed from deterministic-driven to stochastic-dominant processes in response to OCP exposure in soils. The stochastic assembly of viral communities might contribute to the functional redundancy and increased resistance of bacterial hosts in OCP-contaminated soils. By analyzing viromics data, we identified AMGs in the viral genome, but the expression of AMGs within host cells needs to be further explored. This study is the first one that aimed to understand the assembly process of bacterial and viral community under OCP stress. These findings provide information regarding microbial community responses to OCP stress and reveal the collaborative interaction between viral and bacterial communities to resist pollutant stress, thereby revealing a novel avenue for the application of microbiomes to soil remediation.

## MATERIALS AND METHODS

### Sample collection.

Soil samples were collected from a closed organochlorine pesticide factory in Jiangsu Province, China (120.228193′N, 31.758075′E), in 2019. The soil around the organochlorine pesticide factory was exposed continuously to organochlorine pesticides (OCPs), such as chlorobenzene, dichlorobenzene, and nitrochlorobenzene, from 1975 to 2007. Based on the unweighted pair group method using average linkages (UPGMA) clustering result, the nine soils were classified into three distinct groups with no (C1 to C3, no pesticides were detected), light (S1 to S3, the total pesticide content various form 281.3 ± 21.4 to 509.8 ± 28.7 mg · kg^−1^), and heavy (S4 to S6, the total pesticide content various from 1,083.7 ± 40.4 to 4,595.8 ± 344.0 mg · kg^−1^) pesticide stress (11). The surface soil (0 to 20 cm) was collected from each sampling area, and 2.0 kg of soil was selected randomly using the five-point sampling method. Soil samples were briefly stored at 4°C in 1-L sterile polypropylene Falcon tubes and then stored at −80°C until analysis.

### Soil physicochemical properties and pesticide content determination.

Soil samples were ground through a 2.0-mm sieve and then used for the determination of the soil pH, soil organic matter (SOM), cation exchange capacity (CEC), total nitrogen (TN), total phosphorus (TP), and available sulfur (41). The pesticide contents were determined by extracting the pesticides with dichloromethane using an accelerated solvent extraction system (ASE-200; Dionex, USA) followed by gas chromatography-mass spectrometry (GC-MS) analysis (GCMS 6890N-5973 N; Agilent, USA) as described in our previous publications (42, 43).

### Bacterial and viral DNA extraction, sequencing, and analysis.

Bacterial and viral DNA extraction, sequencing, and analysis were performed according to Zheng et al. (11). Briefly, a FastDNA spin kit for soil (MP Bio) and TaKaRa MiniBEST viral RNA/DNA extraction kit 5.0 were used to extract bacterial and viral DNA, respectively. The viral DNA extraction methods are provided in Supplementary Methods in the Supplemental Material. After quality screening was conducted by Cutadapt (v1.2.1), a total of ~8.8 billion clean reads of bacterial metagenomes (~0.8 billion per clean soil samples and ~1.06 billion per pesticide-contaminated soil samples) and ~9.6 billion clean reads (~1.06 billion per sample) of viromes were obtained and used for *de novo* assembly (see Table S6 in the supplemental material) (44, 45). All the raw reads and assembled metagenomics data are publicly accessible and can be download online from https://ngdc.cncb.ac.cn/bioproject/browse/PRJCA003886.

### Viral protein clustering and distribution.

Viral protein clustering and distribution were performed according to Zheng et al. (11). Briefly, identified viral contigs were clustered into virus populations (vOTUs) using ClusterGenomes (v1.1.3; 95% identity and 80% coverage). vOTUs larger than 10 kb were subjected to protein clustering using vConTACT (v2.0; default parameters) based on the NCBI bacterial and archaeal viral RefSeq v85 database (46). All protein sequence alignments were performed using DIAMOND 0.9.10 to group proteins (47). Virus taxonomic annotation was performed using vConTACT (v2.0), and viral proteins were matched to the RefSeq virus database using BLASTp (bitcore, ≥50).

### Viral host prediction.

The following three methods were used for virus host prediction: (i) the tRNA sequences were recovered from the viral genome with ARAGORN (v1.2.38) and then aligned with bacterial sequences by BLAST searches (100% coverage and 100% sequence identity) (48); (ii) the sequence similarity of bacterial and viral CRISPR spacers was used to predict viral hosts. CRISPR spacers were recovered using CRASS from bacterial metagenomic paired-end (PE) reads (49). BLASTn (E value, 10^−10^; 100% nucleotide identity) was used to compare bacterial CRISPR spacer sequences and viral contigs; (iii) viral sequences were submitted to the JGI Virus Sequence Database to predict bacterial hosts (E value, 10^−5^; sequence identity, ≥95%) (48). The tRNA sequence alignment, CRISPR spacers comparison, and JGI database matching linked viral contigs with 35, 28, and 10,869 putative bacterial host sequences, respectively.

### Influence of abiotic factors on taxa and gene composition.

Variance partitioning analysis (VPA) was used to determine the impact of abiotic factors on microbial communities (50). Abiotic factors include soil physicochemical properties (e.g., pH, soil organic matter, and total nitrogen) and pesticide content (e.g., benzene, chlorobenzene, and meta-nitrochlorobenzene) (see Table S7 in the supplemental material). Prior to VPA, bioenv analysis was performed to sort out the effects of combinations of abiotic factors on taxa and genes composition. VPA and bioenv analysis were conducted using the relative abundance of taxa obtained from metagenomic sequencing and genes using the KEGG ontology (KO) group abundance annotated by the KEGG database. The contributions of physicochemical properties and pesticide content to community change were analyzed quantitatively by VPA and bioenv analysis with the “varpart” function and “bioenv” function in the “vegan” package, respectively.

### Community assembly analysis.

The normalized stochasticity ratio (NST) reflects the relative importance of stochasticity in community assembly based on the magnitude of the difference between observed and null expectation as a quantitative measure of stochasticity. The NST value is between 0 and 1. When the NST value of the community is above 50%, stochastic processes are considered dominant in the community, while a value lower than 50% indicates deterministic processes that drive community assembly. The NST can be calculated as follows:

NSS=∑ijξ(Cij,Eij¯)−mink[∑ijξ(Eij(k),Eij¯)]∑ijξ(C Dij,Eij¯)−mink[∑ijξ(Eij(k),Eij¯)],

C Dij={1 Cij≥Eij¯0 Cij<Eij¯,

$$\xi \left(x,\text{y}\right)=\;\frac{x-y}{x-\delta }\;\delta =\{\begin{array}{c} 0\;x\geq y\\ 1\;x<y \end{array},$$

NST=1−NSS,

where *D_ij_* and *C_ij_* are the dissimilarity and actual similarity values between the *i*th community and the *j*th community, respectively; ^*D*^*C_ij_* represents the similarity between community *i* and *j* under extremely deterministic assembly; and *E_ij_^(k)^* indicates one of the null expected values of similarity between community *i* and *j* under stochastic assembly. The “tNST” and “nst.boot” functions of the “NST” package were used to calculate NST values.

Migration rate data were calculated by an analysis of observed operational taxonomic unit (OTU) mean relative abundances using the likelihood formula developed by Tetame with Etienne (34, 51). With this method, the value of the migration rate is between 0 and 1. Higher migration rate values indicate that microbial communities are less limited by dispersal. In other words, when the value of migration approaches 1, the dispersal limitation decreases (i.e., all species migrate from the regional species pool) (34).

### Niche breadth.

We calculated niche breadth to represent the fitness and diversity of available resources of communities. The Levins niche breadth index was calculated using the following function:

$$B_{j}=1/\overset{N}{\underset{i=1}{\sum }}P_{ij^{2}},$$

where *B_j_* is the niche breadth of taxon *j* in the metacommunity, *N* represents the count of communities in the metacommunity, and *P_ij_* refers to the proportion of taxon *j* in community *i*. The niche breadth was calculated using the “nst.boot” functions of the “SPAA” package in R (52).

### Data statistical analysis.

Data were analyzed using R 4.1.2 and visualized with GraphPad Prism 8.0.2. Alpha and beta diversity were calculated using the “vegan” package (53). One-way ANOVA was used to identify significant differences between samples. An NMDS stress value of <0.05 based on the Bray-Curtis distance was considered to represent a good fit of NMDS analysis to the data. ADONIS was performed to determine whether the difference identified by NMDS was significant. Virus and host link networks were visualized with Cytoscape 3.9.1.

## References

[1] He T, Li H, Zhang X. 2017. Deep-sea hydrothermal vent viruses compensate for microbial metabolism in virus-host interactions. mBio 8:e00893-17. 10.1128/mBio.00893-17. 28698277 PMC5513705

[2] Trubl G, Jang HB, Roux S, Emerson JB, Solonenko N, Vik DR, Solden L, Ellenbogen J, Runyon AT, Bolduc B, Woodcroft BJ, Saleska SR, Tyson GW, Wrighton KC, Sullivan MB, Rich VI. 2018. Soil viruses are underexplored players in ecosystem carbon processing. mSystems 3:e00076-18. 10.1128/mSystems.00076-18. 30320215 PMC6172770

[3] Haaber J, Leisner JJ, Cohn MT, Catalan-Moreno A, Nielsen JB, Westh H, Penadés JR, Ingmer H. 2016. Bacterial viruses enable their host to acquire antibiotic resistance genes from neighbouring cells. Nat Commun 7:13333. 10.1038/ncomms13333. 27819286 PMC5103068

[4] Ghosh D, Roy K, Williamson KE, White DC, Wommack KE, Sublette KL, Radosevich M. 2008. Prevalence of lysogeny among soil bacteria and presence of 16S rRNA and trzN genes in viral-community DNA. Appl Environ Microbiol 74:495–502. 10.1128/AEM.01435-07. 17993550 PMC2223242

[5] Adriaenssens EM, Van Zyl L, De Maayer P, Rubagotti E, Rybicki E, Tuffin M, Cowan DA. 2015. Metagenomic analysis of the viral community in Namib Desert hypoliths. Environ Microbiol 17:480–495. 10.1111/1462-2920.12528. 24912085

[6] Emerson JB, Roux S, Brum JR, Bolduc B, Woodcroft BJ, Jang HB, Singleton CM, Solden LM, Naas AE, Boyd JA, Hodgkins SB, Wilson RM, Trubl G, Li C, Frolking S, Pope PB, Wrighton KC, Crill PM, Chanton JP, Saleska SR, Tyson GW, Rich VI, Sullivan MB. 2018. Host-linked soil viral ecology along a permafrost thaw gradient. Nat Microbiol 3:870–880. 10.1038/s41564-018-0190-y. 30013236 PMC6786970

[7] Kuzyakov Y, Mason-Jones K. 2018. Viruses in soil: nano-scale undead drivers of microbial life, biogeochemical turnover and ecosystem functions. Soil Biol Biochem 127:305–317. 10.1016/j.soilbio.2018.09.032.

[8] Brum JR, Sullivan MB. 2015. Rising to the challenge: accelerated pace of discovery transforms marine virology. Nat Rev Microbiol 13:147–159. 10.1038/nrmicro3404. 25639680

[9] Hwang Y, Rahlff J, Schulze-Makuch D, Schloter M, Probst AJ. 2021. Diverse viruses carrying genes for microbial extremotolerance in the Atacama Desert hyperarid soil. mSystems 6:e00385-21. 10.1128/mSystems.00385-21. 34006626 PMC8269230

[10] Bi L, Yu D-T, Du S, Zhang L-M, Zhang L-Y, Wu C-F, Xiong C, Han L-L, He J-Z. 2021. Diversity and potential biogeochemical impacts of viruses in bulk and rhizosphere soils. Environ Microbiol 23:588–599. 10.1111/1462-2920.15010. 32249528

[11] Zheng X, Jahn MT, Sun M, Friman V-P, Balcazar JL, Wang J, Shi Y, Gong X, Hu F, Zhu Y-G. 2022. Organochlorine contamination enriches virus-encoded metabolism and pesticide degradation associated auxiliary genes in soil microbiomes. ISME J 16:1397–1408. 10.1038/s41396-022-01188-w. 35039616 PMC9038774

[12] Huang D, Yu P, Ye M, Schwarz C, Jiang X, Alvarez PJJ. 2021. Enhanced mutualistic symbiosis between soil phages and bacteria with elevated chromium-induced environmental stress. Microbiome 9:150. 10.1186/s40168-021-01074-1. 34183048 PMC8240259

[13] Hubbell SP. 2005. Neutral theory in community ecology and the hypothesis of functional equivalence. Funct Ecol 19:166–172. 10.1111/j.0269-8463.2005.00965.x.

[14] Zhou J, Ning D. 2017. Stochastic community assembly: does it matter in microbial ecology? Microbiol Mol Biol Rev 81:e00002-17. 10.1128/MMBR.00002-17. 29021219 PMC5706748

[15] Jiao S, Chen W, Wei G. 2017. Biogeography and ecological diversity patterns of rare and abundant bacteria in oil-contaminated soils. Mol Ecol 26:5305–5317. 10.1111/mec.14218. 28665016

[16] Qiu L, Fang W, He H, Liang Z, Zhan Y, Lu Q, Liang D, He Z, Mai B, Wang S. 2020. Organohalide-respiring bacteria in polluted urban rivers employ novel bifunctional reductive dehalogenases to dechlorinate polychlorinated biphenyls and tetrachloroethene. Environ Sci Technol 54:8791–8800. 10.1021/acs.est.0c01569. 32551541

[17] Danczak RE, Daly RA, Borton MA, Stegen JC, Roux S, Wrighton KC, Wilkins MJ. 2020. Ecological assembly processes are coordinated between bacterial and viral communities in fractured shale ecosystems. mSystems 5:e00098-20. 10.1128/mSystems.00098-20. 32184367 PMC7380583

[18] Camenzuli L, Scheringer M, Hungerbühler K. 2016. Local organochlorine pesticide concentrations in soil put into a global perspective. Environ Pollut 217:11–18. 10.1016/j.envpol.2015.08.028. 26341663

[19] Wu X, Chen A, Yuan Z, Kang H, Xie Z. 2020. Atmospheric organochlorine pesticides (OCPs) and polychlorinated biphenyls (PCBs) in the Antarctic marginal seas: distribution, sources and transportation. Chemosphere 258:127359. 10.1016/j.chemosphere.2020.127359. 32544807

[20] Souza RC, Portella RB, Almeida PVNB, Pinto CO, Gubert P, Santos da Silva JD, Nakamura TC, do Rego EL. 2020. Human milk contamination by nine organochlorine pesticide residues (OCPs). J Environ Sci Health B 55:530–538. 10.1080/03601234.2020.1729630. 32525731

[21] Gutierrez R, Ortiz R, Vega S, Schettino B, Ramirez ML, Perez JJ. 2013. Residues levels of organochlorine pesticide in cow’s milk from industrial farms in Hidalgo, Mexico. J Environ Sci Health B 48:935–940. 10.1080/03601234.2013.816592. 23998305

[22] Dell'Anno F, Rastelli E, Tangherlini M, Corinaldesi C, Sansone C, Brunet C, Balzano S, Ianora A, Musco L, Montereali MR, Dell'Anno A. 2021. Highly contaminated marine sediments can host rare bacterial taxa potentially useful for bioremediation. Front Microbiol 12:584850. 10.3389/fmicb.2021.584850. 33732217 PMC7956957

[23] Zhu X, Liao C, Song D, Yan X, Wan Y, Sun H, Wang X. 2023. Glucose facilitates the acclimation of organohalide-respiring bacteria. J Hazard Mater 444:130421. 10.1016/j.jhazmat.2022.130421. 36427483

[24] Shen R, Yu L, Xu P, Liang Z, Lu Q, Liang D, He Z, Wang S. 2021. Water content as a primary parameter determines microbial reductive dechlorination activities in soil. Chemosphere 267:129152. 10.1016/j.chemosphere.2020.129152. 33316619

[25] Ning D, Deng Y, Tiedje JM, Zhou J. 2019. A general framework for quantitatively assessing ecological stochasticity. Proc Natl Acad Sci USA 116:16892–16898. 10.1073/pnas.1904623116. 31391302 PMC6708315

[26] McCann KS. 2000. The diversity-stability debate. Nature 405:228–233. 10.1038/35012234. 10821283

[27] Avila-Jimenez M-L, Burns G, He Z, Zhou J, Hodson A, Avila-Jimenez J-L, Pearce D. 2020. Functional associations and resilience in microbial communities. Microorganisms 8:951. 10.3390/microorganisms8060951. 32599781 PMC7357002

[28] Carvalho G, Fouchet D, Danesh G, Godeux A-S, Laaberki M-H, Pontier D, Charpentier X, Venner S. 2020. Bacterial transformation buffers environmental fluctuations through the reversible integration of mobile genetic elements. mBio 11:e02443-19. 10.1128/mBio.02443-19. 32127449 PMC7064763

[29] Dopheide A, Lear G, He Z, Zhou J, Lewis GD. 2015. Functional gene composition, diversity and redundancy in microbial stream biofilm communities. PLoS One 10:e0123179. 10.1371/journal.pone.0123179. 25849814 PMC4388685

[30] Zhao J, Frauenkron-Machedjou VJ, Kardashliev T, Ruff AJ, Zhu L, Bocola M, Schwaneberg U. 2017. Amino acid substitutions in random mutagenesis libraries: lessons from analyzing 3000 mutations. Appl Microbiol Biotechnol 101:3177–3187. 10.1007/s00253-016-8035-1. 28050632

[31] Tamilmaran N, Sankaranarayanan R, Selvakumar A S P, Munavar MH. 2021. Horizontal transfer of domains in ssrA gene among Enterobacteriaceae. Genes Cells 26:541–550. 10.1111/gtc.12869. 33971069

[32] Fargione J, Brown CS, Tilman D. 2003. Community assembly and invasion: an experimental test of neutral versus niche processes. Proc Natl Acad Sci USA 100:8916–8920. 10.1073/pnas.1033107100. 12843401 PMC166413

[33] Xia R, Shi Y, Wang X, Wu Y, Sun M, Hu F. 2022. Metagenomic sequencing reveals that the assembly of functional genes and taxa varied highly and lacked redundancy in the earthworm gut compared with soil under vanadium stress. mSystems 7:e01253-21. 10.1128/mSystems.01253-21. 35089099 PMC8725585

[34] Etienne RS. 2005. A new sampling formula for neutral biodiversity. Ecology Lett 8:253–260. 10.1111/j.1461-0248.2004.00717.x.

[35] Carscadden KA, Emery NC, Arnillas CA, Cadotte MW, Afkhami ME, Gravel D, Livingstone SW, Wiens JJ. 2020. Niche breadth: causes and consequences for ecology, evolution, and conservation. Q Rev Biol 95:179–214. 10.1086/710388.

[36] Verster AJ, Borenstein E. 2018. Competitive lottery-based assembly of selected clades in the human gut microbiome. Microbiome 6:186. 10.1186/s40168-018-0571-8. 30340536 PMC6195700

[37] Tang X, Yu P, Tang L, Zhou M, Fan C, Lu Y, Mathieu J, Xiong W, Alvarez PJ. 2019. Bacteriophages from arsenic-resistant bacteria transduced resistance genes, which changed arsenic speciation and increased soil toxicity. Environ Sci Technol Lett 6:675–680. 10.1021/acs.estlett.9b00600.

[38] Salmond GPC, Fineran PC. 2015. A century of the phage: past, present and future. Nat Rev Microbiol 13:777–786. 10.1038/nrmicro3564. 26548913

[39] González-Villalobos E, Balcázar JL. 2022. Does phage-mediated horizontal gene transfer represent an environmental risk? Trends Microbiol 30:1022–1024. 10.1016/j.tim.2022.07.011. 35970720

[40] Ross J, Topp E. 2015. Abundance of antibiotic resistance genes in bacteriophage following soil fertilization with dairy manure or municipal biosolids, and evidence for potential transduction. Appl Environ Microbiol 81:7905–7913. 10.1128/AEM.02363-15. 26341211 PMC4616940

[41] Jones J. 2018. Soil analysis handbook of reference methods. CRC Press, Boca Raton, FL.

[42] Ye M, Sun M, Hu F, Kengara FO, Jiang X, Luo Y, Yang X. 2014. Remediation of organochlorine pesticides (OCPs) contaminated site by successive methyl-β-cyclodextrin (MCD) and sunflower oil enhanced soil washing—Portulaca oleracea L. cultivation. Chemosphere 105:119–125. 10.1016/j.chemosphere.2013.12.058. 24411840

[43] Sun M, Ye M, Wu J, Feng Y, Wan J, Tian D, Shen F, Liu K, Hu F, Li H, Jiang X, Yang L, Kengara FO. 2015. Positive relationship detected between soil bioaccessible organic pollutants and antibiotic resistance genes at dairy farms in Nanjing, Eastern China. Environ Pollut 206:421–428. 10.1016/j.envpol.2015.07.022. 26256145

[44] Martin M. 2011. Cutadapt removes adapter sequences from high-throughput sequencing reads. EMBnet J 17:10–12. 10.14806/ej.17.1.200.

[45] Li D, Luo R, Liu C-M, Leung C-M, Ting H-F, Sadakane K, Yamashita H, Lam T-W. 2016. MEGAHIT v1.0: a fast and scalable metagenome assembler driven by advanced methodologies and community practices. Methods 102:3–11. 10.1016/j.ymeth.2016.02.020. 27012178

[46] Gregory AC, Zayed AA, Conceição-Neto N, Temperton B, Bolduc B, Alberti A, Ardyna M, Arkhipova K, Carmichael M, Cruaud C, Dimier C, Domínguez-Huerta G, Ferland J, Kandels S, Liu Y, Marec C, Pesant S, Picheral M, Pisarev S, Poulain J, Tremblay J-É, Vik D, Babin M, Bowler C, Culley AI, de Vargas C, Dutilh BE, Iudicone D, Karp-Boss L, Roux S, Sunagawa S, Wincker P, Sullivan MB, Tara Oceans Coordinators. 2019. Marine DNA viral macro- and microdiversity from pole to pole. Cell 177:1109–1123.e14. 10.1016/j.cell.2019.03.040. 31031001 PMC6525058

[47] Buchfink B, Xie C, Huson DH. 2015. Fast and sensitive protein alignment using DIAMOND. Nat Methods 12:59–60. 10.1038/nmeth.3176. 25402007

[48] Paez-Espino D, Eloe-Fadrosh EA, Pavlopoulos GA, Thomas AD, Huntemann M, Mikhailova N, Rubin E, Ivanova NN, Kyrpides NC. 2016. Uncovering Earth’s virome. Nature 536:425–430. 10.1038/nature19094. 27533034

[49] Skennerton CT, Imelfort M, Tyson GW. 2013. Crass: identification and reconstruction of CRISPR from unassembled metagenomic data. Nucleic Acids Res 41:e105. 10.1093/nar/gkt183. 23511966 PMC3664793

[50] Xun W, Li W, Xiong W, Ren Y, Liu Y, Miao Y, Xu Z, Zhang N, Shen Q, Zhang R. 2019. Diversity-triggered deterministic bacterial assembly constrains community functions. Nat Commun 10:3833. 10.1038/s41467-019-11787-5. 31444343 PMC6707308

[51] Dumbrell AJ, Nelson M, Helgason T, Dytham C, Fitter AH. 2010. Relative roles of niche and neutral processes in structuring a soil microbial community. ISME J 4:337–345. 10.1038/ismej.2009.122. 19924158

[52] Xiong C, He J-Z, Singh BK, Zhu Y-G, Wang J-T, Li P-P, Zhang Q-B, Han L-L, Shen J-P, Ge A-H, Wu C-F, Zhang L-M. 2021. Rare taxa maintain the stability of crop mycobiomes and ecosystem functions. Environ Microbiol 23:1907–1924. 10.1111/1462-2920.15262. 32996254

[53] Knight R, Vrbanac A, Taylor BC, Aksenov A, Callewaert C, Debelius J, Gonzalez A, Kosciolek T, McCall L-I, McDonald D, Melnik AV, Morton JT, Navas J, Quinn RA, Sanders JG, Swafford AD, Thompson LR, Tripathi A, Xu ZZ, Zaneveld JR, Zhu Q, Caporaso JG, Dorrestein PC. 2018. Best practices for analysing microbiomes. Nat Rev Microbiol 16:410–422. 10.1038/s41579-018-0029-9. 29795328

