# Supplementary material for: Viral and Bacterial Communities Collaborate through Complementary Assembly Processes in Soil to Survive Organochlorine Contamination
Source: Appl Environ Microbiol. 2023 Feb 21;89(3):e01810-22. doi: 10.1128/aem.01810-22 (PMC10056961; doi:10.1128/aem.01810-22)
Supplement: Supplemental file 2 — Supplemental material. Download aem.01810-22-s0002.pdf, PDF file, 0.6 MB [file aem.01810-22-s0002.pdf]

Supplementary methods

Bacterial and viral DNA extraction, sequencing, and analysis

Bacterial DNA extraction, metagenomic sequencing, and analysis were performed according to Zheng et al. (1). Briefly, bacterial DNA was extracted using the FastDNA Spin kit for soil (MP Bio, Solon, OH, USA) following the manufacturer's instructions. The viral DNA extraction methods are as follows (2, 3): to separate viral DNA from bacterial DNA, suspending soil samples in potassium citrate buffer (1.92 $g \cdot L^{-1}$ $Na_2HPO_4 \cdot 12H_2O$, 10 $g \cdot L^{-1}$ $C_6H_5K_3O_7$ and 0.24 $g \cdot L^{-1}$ KH_2PO_4; pH = 7) at 4°C for 15 min. The mixture was sonicated (100W, 47kHz, 3min) on ice, and then was centrifuged (7000 rpm, 10 min) to obtain a supernatant containing commixtures and virus particles. The supernatant was centrifuged again (7000 rpm, 15 min) and filtered to remove commixtures larger than 0.22 μm. The virus particles were enriched by using tangential flow filtration technology (TFF, Sartorius Vivaflow50 30,000 MWCO PES, USA). The obtained viral particles were treated with a DNase I:RNase A (37°C; 30 min) mixture in 2:1 to remove free DNA and RNA fragments. Viral DNA was extracted using Takara MiniBEST Viral RNA/DNA Extraction Kit 5.0, and the viral DNA was performed to whole genome amplification (KAPA HiFi HotStart ReadyMix).

The amplification viral DNA and bacterial DNA were subjected to metagenomic sequencing, and each library of 400 bp insert-size fragments was used to generate 150 bp paired-end reads using the HiSeq 4000 platform (Illumina, San Diego, CA, USA). The quality control of raw reads of bacteria and viruses ware performed using Cutadapt 1.2.1, followed by *de novo* assembly using Megahit 1.2.6 (k-mer~ parameter settings)

(4, 5). Open reading frames (ORFs) were predicted using MetaGeneAnnotator (6), and then non-redundant sequences were constructed using CD-HIT (90% sequencing similarity and 90 % coverage) (7). Bacterial taxonomy at species levels were annotated using the lowest common ancestor (LCA) algorithm in MEGAN 5 through the NCBI-NT reference database (8). Viral contigs were identified using VirSorter 1.0.5, and viral sequences >5 kb were further filtered using the vhmm pipeline (9). KEGG (kobas3.0.3) and CAZyme (cazydb.07312018.fa) databases were used to annotate non-redundant proteins of bacterial and viral genomes (10, 11). The possible AMGs in viral gene sequences were identified by DRAM-V with default parameters as previously described (1).

Reference:

1. Zheng X, Jahn MT, Sun M, Friman V-P, Balcazar JL, Wang J, Shi Y, Gong X, Hu F, Zhu Y-G. 2022. Organochlorine contamination enriches virus-encoded metabolism and pesticide degradation associated auxiliary genes in soil microbiomes. ISME J 16:1397–1408.

2. Trubl G, Roux S, Solonenko N, Li Y-F, Bolduc B, Rodríguez-Ramos J, Eloe-Fadrosh EA, Rich VI, Sullivan MB. 2019. Towards optimized viral metagenomes for double-stranded and single-stranded DNA viruses from challenging soils. PeerJ 7:e7265.

3. Adriaenssens EM, Kramer R, Van Goethem MW, Makhalanyane TP, Hogg I,

Cowan DA. 2017. Environmental drivers of viral community composition in Antarctic soils identified by viromics. Microbiome 5:83.

4. Martin M. 2011. Cutadapt removes adapter sequences from high-throughput sequencing reads. 1. EMBnet.journal 17:10–12.

5. Li D, Luo R, Liu C-M, Leung C-M, Ting H-F, Sadakane K, Yamashita H, Lam T-W. 2016. MEGAHIT v1.0: A fast and scalable metagenome assembler driven by advanced methodologies and community practices. Methods 102:3–11.

6. Noguchi H, Taniguchi T, Itoh T. 2008. MetaGeneAnnotator: detecting species-specific patterns of ribosomal binding site for precise gene prediction in anonymous prokaryotic and phage genomes. DNA Res 15:387–396.

7. Li W, Godzik A. 2006. Cd-hit: a fast program for clustering and comparing large sets of protein or nucleotide sequences. Bioinformatics 22:1658–1659.

8. Huson DH, Mitra S, Ruscheweyh H-J, Weber N, Schuster SC. 2011. Integrative analysis of environmental sequences using MEGAN4. Genome Res 21:1552–1560.

9. Roux S, Enault F, Hurwitz BL, Sullivan MB. 2015. VirSorter: mining viral signal from microbial genomic data. PeerJ 3:e985.

10. Kanehisa M, Sato Y, Kawashima M, Furumichi M, Tanabe M. 2016. KEGG as a reference resource for gene and protein annotation. Nucleic Acids Res 44:D457-462.

11. Yin Y, Mao X, Yang J, Chen X, Mao F, Xu Y. 2012. dbCAN: a web resource for automated carbohydrate-active enzyme annotation. Nucleic Acids Res 40:W445-451.

Supplementary figures

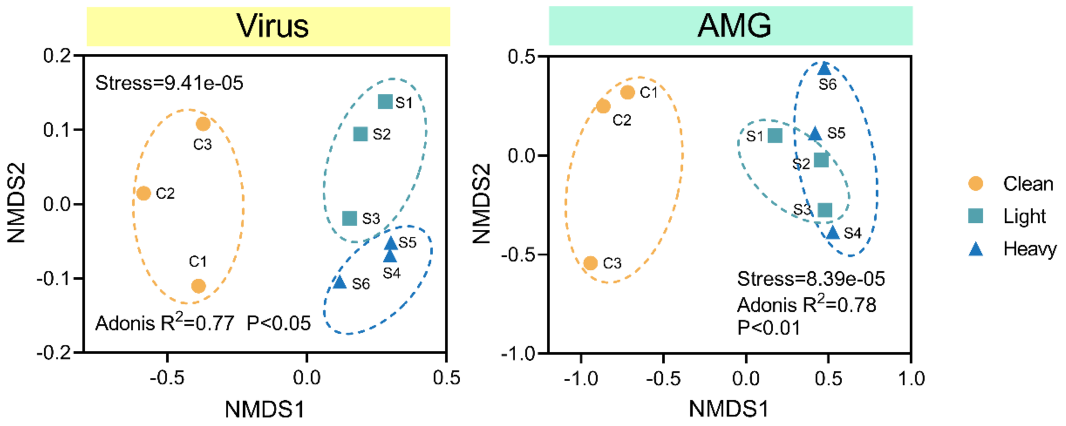

Supplementary Fig. S1. NMDS analysis of virus taxa and AMGs.

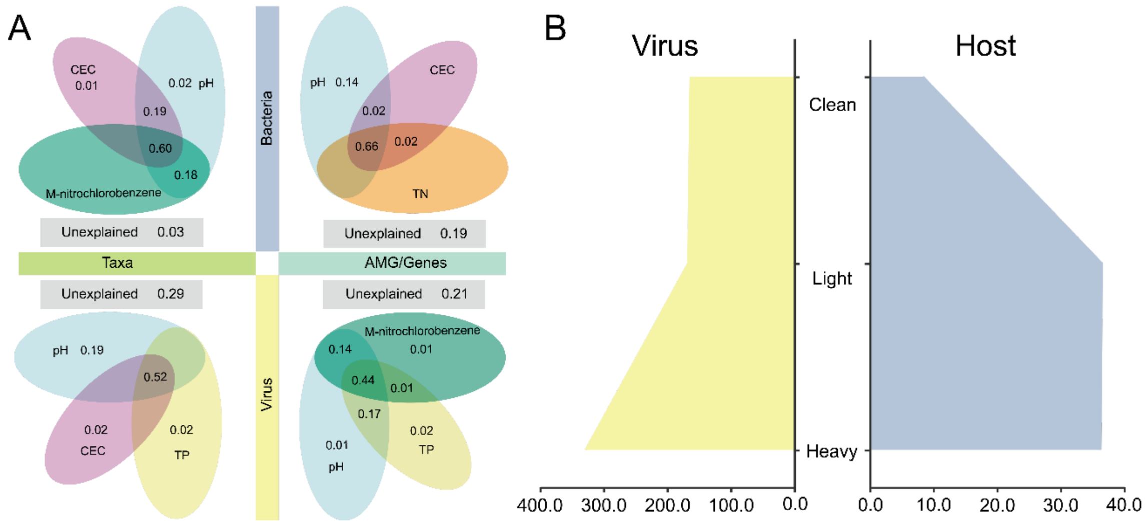

Supplementary Fig. S2. (A) Variation partitioning analysis (VPA) to indicate the impact of environmental factors on bacterial and viral taxa and functional genes. Venn diagram show the degree of separate and joint explanation of variation in bacterial and viral taxa and functional gene composition by environmental factors. Values < 0 are not shown. TP: total phosphorus; TN: total nitrogen; CEC: cation exchange capacity; m-nitrochlorobenzene; pH. **(B)** Differences in niche breadth of virus and host in clean and OCP-contaminated soil.
